# A predictive model for cytarabine syndrome-associated febrile episodes in children with acute lymphoblastic leukemia

**DOI:** 10.3389/fped.2026.1957986

**Published:** 2026-10-07

**Authors:** Zheng Wu, Lixiang Zhu, Yi Liu, Fangyuan Lai, Zebin Chen, Feiqiu Wen, Huirong Mai, Xuejuan Li

**Affiliations:** 1 Department of Pharmacy, Shenzhen Children's Hospital, Shenzhen, China; 2 Department of Hematology and Oncology, Shenzhen Children's Hospital, Shenzhen, China

**Keywords:** acute lymphoblastic leukemia, children, cytarabine syndrome, fever, predictive model

## Abstract

**Objective:**

This study aims to develop a predictive model for cytarabine syndrome (CS)-related fever in pediatric patients with acute lymphoblastic leukemia (ALL). By integrating clinical features and laboratory indicators, we seek to establish an evidence-based diagnostic tool to assist clinicians in decision-making and optimize the use of antimicrobial agents.

**Methods:**

Study Design: A retrospective case-control study was conducted involving 72 ALL children (32 in the CS group vs. 40 in the infection group) who developed fever (≥37.5 °C) following cytarabine treatment. Model Development: Multivariate logistic regression was used to identify independent predictors. A nomogram was constructed. The model's discriminative ability was evaluated using the receiver operating characteristic (ROC) curve, calibration was assessed via the Hosmer-Lemeshow test, and clinical utility was validated using decision curve analysis (DCA).

**Results:**

Key Predictors: Multivariate analysis revealed that BSA (OR = 0.01, 95% CI: 0.00–0.17) and hs-CRP (OR = 0.84, 95% CI: 0.72–0.97) were the most significant predictors of CS. Model Performance: The ROC analysis demonstrated an AUC of 0.785, with internal validation via 1000 bootstrap resamples yielding a 95% confidence interval of 0.675–0.882. The calibration curve demonstrated excellent agreement between predicted and observed outcomes (MAE = 0.039). Decision curve analysis indicated that the model provided superior net benefits across most threshold probabilities (0.2–0.8) compared to strategies of universal antibiotic use or no intervention.

**Conclusion:**

The developed predictive model demonstrates high discriminative performance and robust clinical applicability, functioning as an effective quantitative instrument for early identification of cytarabine syndrome (CS) and facilitating evidence-based antibiotic stewardship.

## Introduction

Cytarabine (Ara-C) is a nucleoside antimetabolite widely used in the treatment of acute lymphoblastic leukemia (ALL) and other hematologic malignancies (1–4). It exerts its antitumor effect by inhibiting DNA synthesis. However, its clinical application is often accompanied by a distinct adverse drug reaction—cytarabine syndrome (CS) (5–8). CS is characterized by a classic triad of clinical manifestations: fever (≥37.5 °C), rash, and arthralgia. Its pathophysiological mechanism may involve abnormal release of cytokines such as IL-6 and TNF-α (7, 9, 10). In the treatment of childhood ALL, distinguishing between cytarabine syndrome (CS)-related fever and infectious fever remains particularly challenging especially during the post-chemotherapy myelosuppression phase, when patients are often immunocompromised (11–17).

Two major issues currently exist in clinical practice: First, there is a significant lack of information in the drug labeling. Although the package insert indicates that CS occurs in ≥10% of adults, reliable epidemiological data in pediatric ALL patients are lacking. Second, diagnosing CS often relies on physician's empirical judgment. Data from our center show that 62.5% (20/32) of children with CS-related fever were misdiagnosed as infections and received antibiotic therapy, highlighting the limitations of the existing labeling guidance (18).

As such, a retrospective analysis was conducted based on clinical data from 72 pediatric ALL patients who developed fever following cytarabine administration. This study established, for the first time, a discriminative diagnostic model specifically tailored for cytarabine syndrome (CS) within the pediatric population. The research focuses on analyzing key clinical parameters such as BSA and inflammatory markers, with the aim of establishing a rapid and reliable differential diagnosis model. By providing clinicians with a decision-support tool, it seeks to advance fever management and promote the rational use of antibiotics.

## Methods

### Study design and participants

This retrospective case-control study was conducted in pediatric patients at the Department of Hematology, Shenzhen Children's Hospital who were diagnosed with acute lymphoblastic leukemia (ALL) and had received cytarabine-containing chemotherapy regimens between April 2021 and May 2025. Each of the participants in the study provided informed consent before enrolment. The study was approved with the protocol number 2024119.

A total of 358 patients were reviewed of which 75 developed fever following cytarabine chemotherapy. After strict screening, three cases were excluded due to indistinguishable fever etiology (e.g., concurrent CS and infection). Thus, 72 children with post-cytarabine fever were included in the final analysis. The standard induction regimen consisted of cytarabine 50 mg/m^2^ administered intravenously every 12 h.

Patients were included if they met the following criteria: (1) age between 1 and 14 years; (2) diagnosis of acute lymphoblastic leukemia (ALL) according to established clinical and pathological criteria; (3) development of fever, defined as an axillary temperature ≥37.5 °C sustained for at least 30 min following chemotherapy; and (4) cytarabine exposure within the period of fever. Exclusion criteria comprised: (1) diagnosis of mature B-cell ALL or mixed-phenotype acute leukemia; (2) presence of clinically or microbiologically confirmed infection such as pulmonary infection or catheter-related bloodstream infection within 48 h preceding fever onset; (3) fever attributed to confirmed viral or other infectious etiologies; (4) comorbid autoimmune disorders or additional malignant neoplasms; (5) incomplete medical records; and (6) cases in which the etiology of fever (i.e., cytarabine syndrome versus infectious fever) could not be conclusively determined.

The following data were extracted from the hospital's electronic medical record system:

(1) Baseline characteristics: age, sex, weight, and other demographic information; (2) Treatment parameters: cytarabine dosage and treatment course; (3) Clinical indicators: time to fever onset post cytarabine administration; (4) Laboratory tests: complete blood count (white blood cell count, absolute neutrophil count, etc.), inflammatory markers (hs-CRP, PCT), and liver/kidney function tests (alanine aminotransferase, creatinine, urea).

### Statistical Analysis

Statistical analysis was conducted using R software (version 4.5.0). Normality of continuous variables was assessed with the Shapiro–Wilk test. Normally distributed variables were expressed as mean ± standard deviation and compared using the independent samples *t*-test. Non-normally distributed variables were reported as median with interquartile range (Q₁, Q₃) and analyzed using the Mann–Whitney U test. Categorical variables were summarized as frequency counts and percentages, with group comparisons performed by chi-square test. A two-sided *p*-value < 0.05 was defined as statistically significant.

Potential predictors associated with cytarabine syndrome were initially screened through univariate analysis, with a significance threshold of *P* < 0.05 applied for variable inclusion. Independent predictors were subsequently identified using multivariate binary logistic regression with stepwise selection, and the results were reported as odds ratios (OR) along with their corresponding 95% confidence intervals (CI). A nomogram was developed to visually represent the multivariate model, delineating the relative contribution of each predictor and its influence on the final risk probability. The discriminative performance of the predictive model was evaluated using receiver operating characteristic (ROC) analysis. To assess the optimism of the model and obtain robust estimates of discriminatory performance, internal validation was performed using 1000 bootstrap resamples. Calibration curves, generated from 1000 bootstrap resamples, were utilized to evaluate the concordance between predicted probabilities and observed outcomes. Finally, decision curve analysis (DCA) was implemented to quantify the clinical net benefit and utility of the predictive model.

## Results

A cohort of 72 pediatric patients diagnosed with acute lymphoblastic leukemia (ALL) who exhibited fever following cytarabine administration. Based on comprehensive diagnostic evaluation, participants were stratified into two distinct groups: a cytarabine syndrome (CS) cohort (*n* = 32, 44.0%) and an infection-associated febrile cohort (*n* = 40, 56.0%).

A comparative evaluation of baseline antibiotic prescribing practices demonstrated statistically significant patterns between the two cohorts: (1). Antibiotic Therapy use: Monotherapeutic strategies predominated within the CS cohort (59.4%, 19/32), with cefoperazone-sulbactam emerging as the most frequently administered antimicrobial agent (73.7%, 14/19). Notably, combination therapy was implemented in merely a single case (3.1%), underscoring the preferential utilization of monotherapy within this patient subset. Conversely, the infection group demonstrated substantially higher utilization of combination regimens, including two-drug (30.0%), three-drug (12.5%), and four-drug (10.0%) therapies, alongside increased use of broad-spectrum antimicrobials such as meropenem and vancomycin. (2). Baseline Characteristic Homogeneity: Demographic, therapeutic, and laboratory parameters exhibited no statistically significant intergroup differences (all *P* > 0.05; Table 1):1) Demographic characteristics: sex *(P* = 0.267) and risk stratification (*P* = 0.204); 2) Treatment parameters: No statistically significant intergroup difference was observed in the time from drug to fever (*P* = 0.116); 3) Laboratory parameters: No statistically significant intergroup differences were observed in albumin (*P* = 0.856), creatinine (*P* = 0.063), leukocyte count (*P* = 0.618), absolute neutrophil count (*P* = 0.937), procalcitonin (*P* = 0.730), alanine aminotransferase (*P* = 0.544), total bilirubin (*P* = 0.200), and urea (*P* = 0.537).

**Table 1:** General characteristics of patients in the Two groups.

Characteristic	Infection Group (*n* = 40)	CS Group (*n* = 32)	Statistic	*P*-value
Albumin (g/L), mean ± SD	34.63 ± 4.96	34.85 ± 5.43	*t* = −0.18	0.856
Creatinine (μmol/L), mean ± SD	25.83 ± 8.40	22.36 ± 6.87	*t* = 1.89	0.063
Age (years), median (IQR)	4.17 (2.90, 6.12)	3.29 (1.61, 4.77)	*Z* = −2.13	0.034
BSA(m^2^), median (IQR)	0.76 (0.59, 0.89)	0.60 (0.51, 0.72)	*Z* = −2.75	0.006
WBC count ( × 10⁹/L), median (IQR)	2.27 (1.50, 3.37)	2.21 (0.92, 3.16)	*Z* = −0.50	0.618
ANC (×10⁹/L), median (IQR)	1.35 (0.56, 1.90)	1.48 (0.52, 1.91)	*Z* = −0.08	0.937
hs-CRP (mg/L), median (IQR)	2.65 (0.78, 9.35)	1.02 (0.30, 2.48)	*Z* = −2.87	0.004
PCT (ng/mL), median (IQR)	0.15 (0.07, 0.30)	0.12 (0.07, 0.31)	*Z* = −0.35	0.730
ALT (IU/L), median (IQR)	31.00 (18.50, 50.75)	30.00 (20.00, 78.00)	*Z* = −0.61	0.544
AST (IU/L), median (IQR)	21.00 (17.75, 30.50)	26.00 (21.50, 32.50)	*Z* = −2.01	0.045
Total bilirubin (μmol/L), median (IQR)	14.10 (8.92, 19.70)	13.70 (7.00, 16.97)	*Z* = −1.28	0.200
Urea (mmol/L), median (IQR)	5.65 (4.33, 7.23)	5.85 (5.02, 7.25)	*Z* = −0.62	0.537
Time from drug to fever (days), median (IQR)	5.00 (3.00, 6.00)	4.00 (2.00, 5.00)	*Z* = −1.57	0.116
Gender, *n* (%)			*χ^2^* = 1.23	0.267
Female	16 (40.00)	17 (53.12)		
Male	24 (60.00)	15 (46.88)		
Risk stratification, *n* (%)			*χ^2^* = 1.61	0.204
Intermediate risk	19 (47.50)	20 (62.50)		
Low risk	21 (52.50)	12 (37.50)		

SD, standard deviation; IQR, interquartile range; WBC, white blood cell; ANC, absolute neutrophil count; hs-CRP, high-sensitivity C-reactive protein; PCT, procalcitonin; ALT, alanine aminotransferase; AST, aspartate aminotransferase; Time from drug to fever: the interval from the end of cytarabine administration to the first documented fever in the nursing system; Statistical tests: t, Student's *t*-test; Z, Mann–Whitney U test; χ^2^, chi-square test.

Comparative analysis using Student's *t*-test and the Mann–Whitney U test identified several statistically significant differences between the cytarabine syndrome (CS) and infection groups: (1) Demographic Parameters: The infection group demonstrated elevated Body Surface Area(BSA) (0.76 m^2^ [IQR 0.59, 0.89]vs. 0.60 m^2^ [IQR 0.51, 0.72]; *P* = 0.006), advanced median age (4.17 years [IQR 2.90–6.12] vs. 3.29 years [IQR 1.61–4.77];*P* = 0.034) relative to the CS cohort. (2) Biomarkers: high-sensitivity C-reactive protein concentrations were significantly elevated in the infection group (hs-CRP) (2.65 mg/L [IQR 0.78–9.35] vs. 1.02 mg/L [IQR 0.30–2.48]; *P* = 0.004); aspartate aminotransferase (AST) activity demonstrated a statistically reduction in this cohort (21.00 IU/L [IQR 17.75–30.50] vs. 26.00 IU/L [IQR 21.50–32.50]; *P* = 0.045). A comprehensive synthesis of these findings is systematically detailed in Table 1.

Univariate and multivariate logistic regression analysis, incorporating variable with univariate significance (*P* < 0.05), established BSA and hs-CRP as independent predictors of cytarabine syndrome (CS) (Table 2). The final model yielded the following quantitative relationships: Each 1 m^2^ increase in BSA was associated with 99% lower odds of CS development (OR = 0.01, 95% CI: 0.00–0.17, *P* = 0.003), while each 1 mg/L increase in hs-CRP was associated with 16% lower odds of CS development (OR = 0.84, 95% CI: 0.72–0.97, *P* = 0.018).

**Table 2:** Analysis of factors influencing cytarabine syndrome (CS)-related fever.

Factor	Univariate OR (95% CI, *P*-value)	Multivariate OR (95% CI, *P*-value)	Final Model OR (95% CI, *P*-value)
Age	0.82 (0.67–1.01, *P* = .063)		
Gender	0.59 (0.23–1.50, *P* = .268)		
BSA	**0.01 (0.00–0.23, *P*** **=** **.004)**	**0.01 (0.00–0.17, *P*** **=** **.003)**	**0.01 (0.00–0.17, *P*** **=** **.003)**
Risk stratification	0.54 (0.21–1.40, *P* = .206)		
WBC count	0.97 (0.72–1.31, *P* = .868)		
ANC	0.95 (0.65–1.39, *P* = .797)		
hs-CRP	**0.83 (0.72–0.97, *P*** **=** **.020)**	**0.84 (0.72–0.97, *P*** **=** **.018)**	**0.84 (0.72–0.97, *P*** **=** **.018)**
PCT	1.18 (0.68–2.04, *P* = .559)		
ALT	1.00 (1.00–1.01, *P* = .249)		
AST	1.03 (0.99–1.07, *P* = .093)		
Total bilirubin	0.95 (0.90–1.01, *P* = .101)		
Albumin	1.01 (0.92–1.10, *P* = .854)		
Creatinine	0.94 (0.88–1.00, *P* = .070)		
Urea	0.98 (0.93–1.04, *P* = .541)		
Time from drug to fever	0.80 (0.61–1.05, *P* = .105)		

Bold values indicate variables retained in the final multivariate model (*P* < 0.05).

OR, odds ratio; CI, confidence interval; WBC, white blood cell; ANC, absolute neutrophil count; hs-CRP, high-sensitivity C-reactive protein; PCT, procalcitonin; ALT, alanine aminotransferase; AST, aspartate aminotransferase.

Nomogram analysis (Figure 1) indicated that BSA and hs-CRP were significantly associated with CS. The total predictive score derived from the model ranged from 80 to 200 points, corresponding to an estimated probability of CS. For instance, a representative patient with a BSA of 0.57 m^2^ and hs-CRP of 0.5 mg/L would be assigned 179 points. Subsequent model conversion indicated a corresponding CS probability of 72.2%.

**Figure 1:**
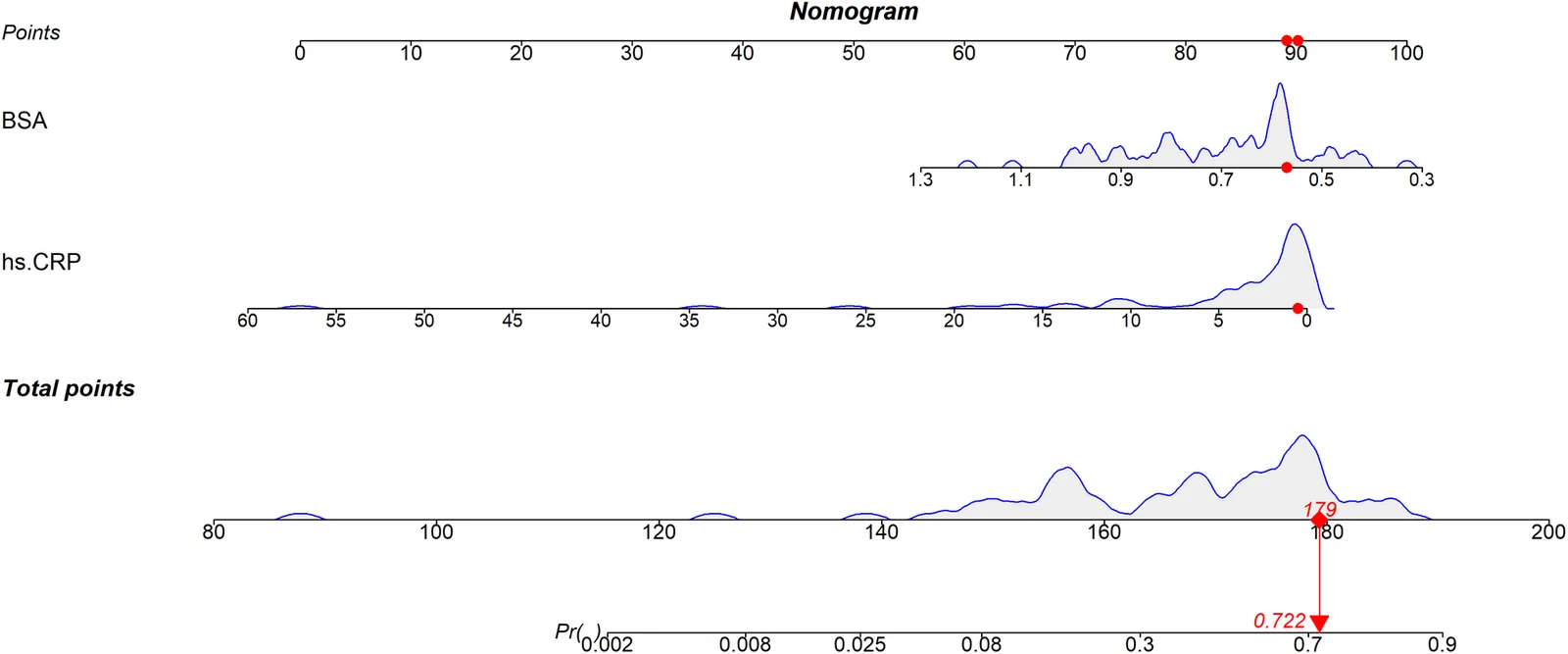
A constructed nomogram for prognostic prediction of a patient with cytarabine. The patient's BSA was 0.57 m^2^ and hs-CRP was 0.5 mg/L. To use the nomogram, the specific points (blackdots) of individual patients are located on each variable axis. Red lines and dots are drawn upward to determine the points received by each variable; the sum (179) of these points is located on the Total Points axis, and a line is drawn downward to the probability axes to determine the probability Cytarabine Syndrome (72.2%).

The discriminatory performance of the model was assessed in the test cohort via receiver operating characteristic (ROC) analysis (Figure 2), which demonstrated an area under the curve (AUC) of 0.785. Internal validation with 1000 bootstrap resamples yielded a 95% confidence interval of 0.675–0.882, reflecting good discriminative capability. Calibration performance was evaluated by comparing bias-corrected predicted probabilities against observed outcomes (Figure 3), showing close alignment with the ideal calibration line. Quantitative metrics further supported model robustness, with a mean absolute error (MAE) of 0.039, a Brier score of 0.1843, and a non-significant Hosmer-Lemeshow test result (*P* = 0.1325). Collectively, these findings indicate that the model exhibits strong discriminative power and calibration accuracy and supports individualized risk assessment of cytarabine syndrome.

**Figure 2:**
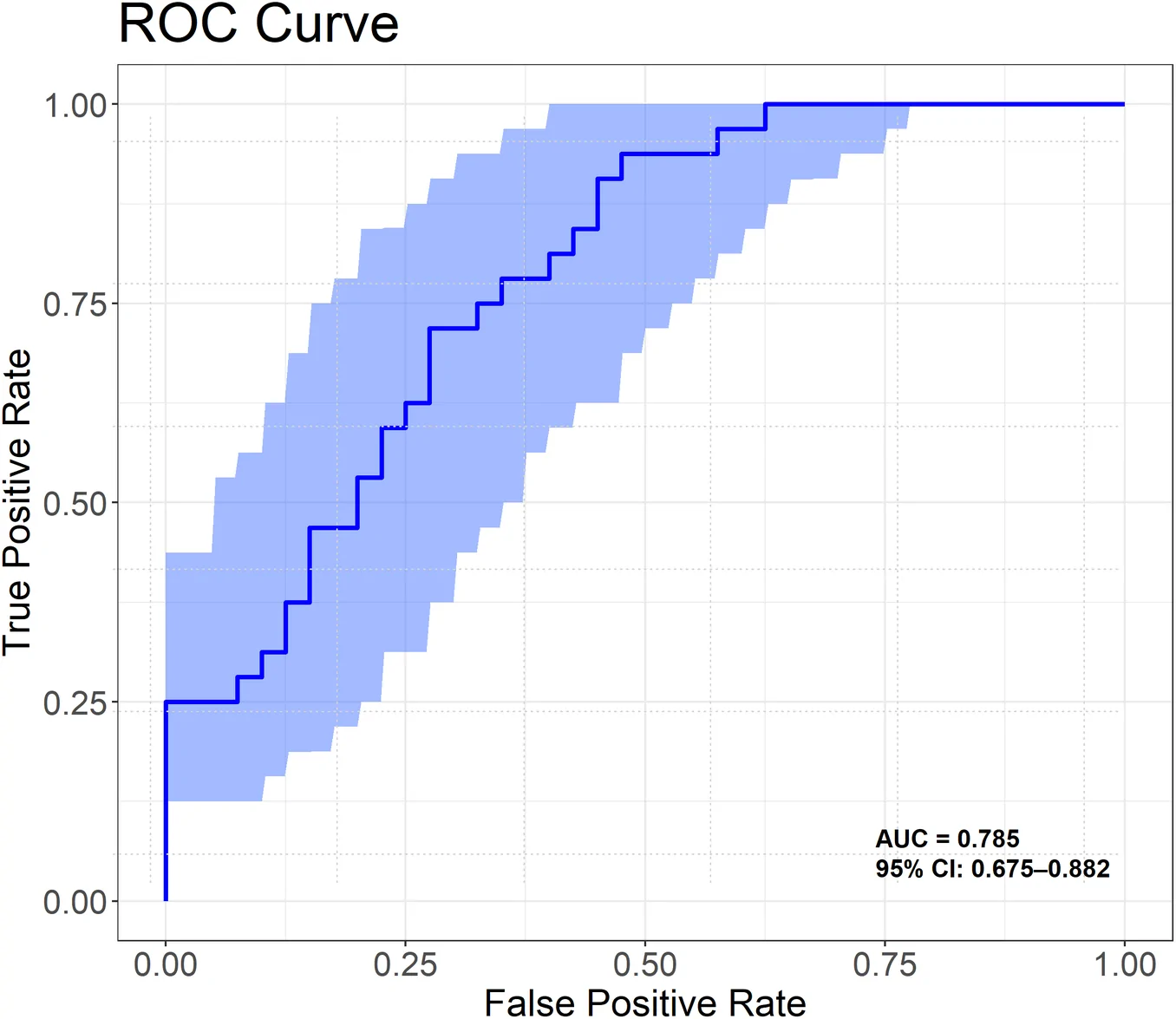
ROC Curve Analysis of the Model with 1000 Bootstrap Resamples. The light blue shaded band around the curve represents the 95% confidence interval derived from 1000 bootstrap resamples. The area under the curve (AUC) was 0.785, with a bootstrap-based 95% confidence interval of 0.675–0.882.

**Figure 3:**
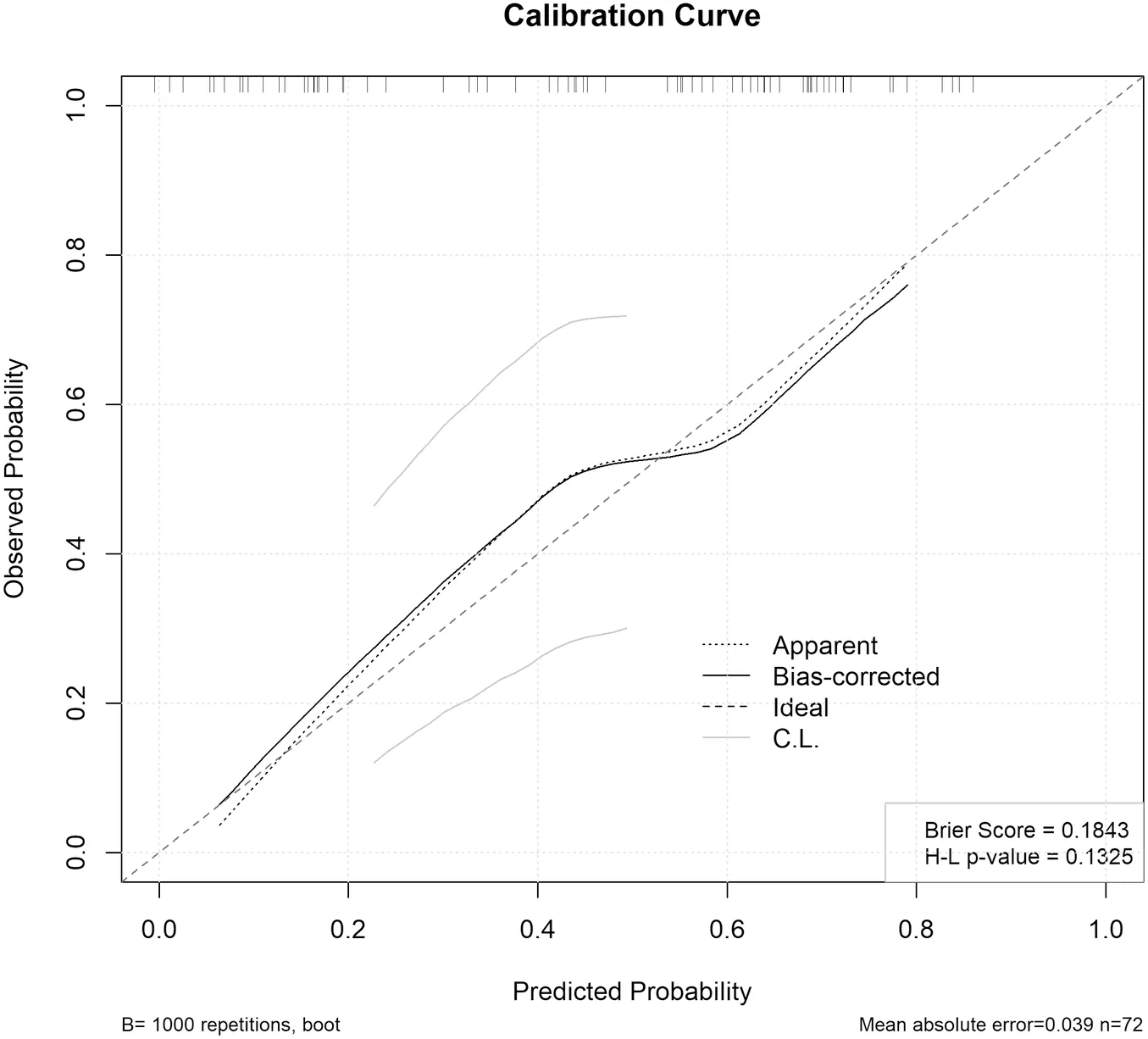
Calibration Curve for Survival Rate Predicted by the Nomogram. The solid line (Bias-corrected) represents the calibration curve corrected by 1000 bootstrap resamples; The dashed line (Apparent) indicates the uncorrected apparent prediction; The dotted diagonal line (45° line) corresponds to the ideal calibration line; The model's mean absolute error (MAE) = 0.039, sample size *n* = 72; Brier Score = 0.1843, and Hosmer-Lemeshow test *P*-value = 0.1325.

Decision curve analysis (DCA) revealed that across the majority of threshold probabilities (0.2–0.8), the proposed predictive model (red curve) yielded superior net benefit compared to both the universal antibiotic administration strategy (gray curve) and the no-intervention approach (black curve) (Figure 4). The model demonstrated optimal clinical utility within the intermediate-to-high threshold probability range (0.4–0.6), which corresponds to a cost-benefit ratio between 2:3 and 3:2. In contrast, at elevated threshold probabilities (>0.8), the net benefit derived from the model diminished rapidly, approaching null value. It is noteworthy that the model sustained a positive net benefit even at stringent threshold probabilities, underscoring its robustness in discriminating high-risk subpopulations.

**Figure 4:**
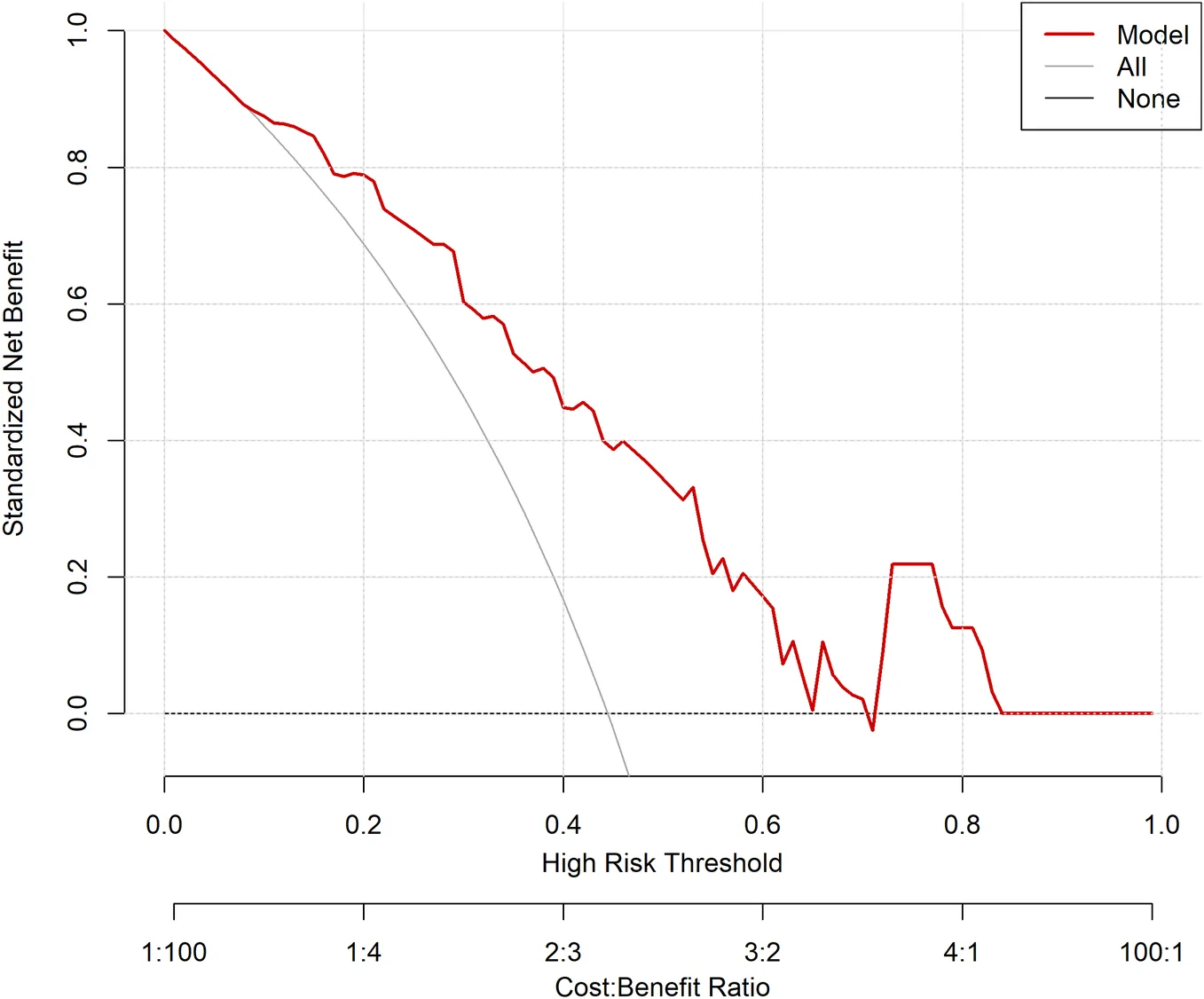
Decision Curve Analysis. The “All” strategy (gray curve) denotes the net benefit resulting from the hypothetical scenario wherein all patients receive the intervention; The “None” strategy (black curve) reflects the net benefit under the assumption that no intervention is administered to any patient.; The “Model” strategy (red curve) illustrates the net benefit achieved when clinical decision-making is informed by the application of the proposed predictive model.

## Discussion

In the study, patients received concurrent empiric antibiotics yet were still adjudicated as cytarabine syndrome on the basis of the characteristic clinical phenotype. Concurrent empiric antibiotics do not constitute an exclusion criterion for CS. Hoover et al. observed febrile reactions in nearly 40% of HD-AraC infusion episodes, all covered with empiric vancomycin, with no confirmed bacteremia in any case; Tay et al. further characterized the classic CS phenotype—symmetric erythematous rash, fever onset 6–12 h after dosing, myalgia and conjunctival injection, and prompt defervescence upon glucocorticoid administration—none of which was obscured by simultaneous antibacterial use (19, 20).

This study identified BSA as an independent protective factor against cytarabine-associated fever (OR 0.01, 95% CI 0.00–0.17), indicating that children with smaller BSA are at substantially higher risk of developing cytarabine fever. Standard cytarabine regimens are BSA-standardized, such that BSA directly governs the absolute exposure delivered. Cytarabine is rapidly deaminated by cytidine deaminase to the inactive metabolite ara-U, which is predominantly excreted renally (21). In pediatric cohorts, smaller BSA is intrinsically correlated with younger age, a period during which the developmental immaturity of drug elimination pathways may further constrain cytarabine clearance (22), potentially prolonging systemic exposure. Population pharmacokinetic analyses in pediatric patients further reveal that BSA is a significant allometric covariate on cytarabine clearance, clearance increases proportionally with BSA, and smaller-BSA patients retain relatively lower clearance capacity per unit of administered dose (23).

We therefore postulate that in smaller-BSA children, the convergence of lower BSA-scaled clearance and developmentally immature drug disposition yields intensified per-tissue cytarabine exposure, which in turn lowers the threshold for cytokine-mediated febrile response. This perspective is supported by existing literature: T Ek et al. demonstrated that high-dose cytarabine can induce TNF-α release, followed by a cascade of other pro-inflammatory cytokines, which are likely responsible for the clinical manifestations of cytarabine syndrome (9). Similarly, Xiao-Jun Xu et al. reported that IL-6 and IL-10 exhibit high predictive value for identifying patients at risk of severe febrile complications (10). Furthermore, H Schönbohn et al. observed significantly elevated IL-8 levels in acute myeloid leukemia patients with febrile neutropenia and/or severe infections (24). Synthesizing these lines of evidence, we infer that children with greater BSA—by virtue of higher allometric clearance and correspondingly attenuated per-tissue cytarabine exposure—are less likely to surpass the threshold for cytokine-mediated febrile response, thereby conferring a lower observed risk of cytarabine syndrome–associated fever in the present model.

Regarding hs-CRP, previous studies have consistently demonstrated a positive correlation between elevated hs-CRP levels and increased infection risk. This finding aligns with the results of the present study. Specifically, Grützmeier et al. reported that during 44 episodes of bacterial or fungal infection, CRP increased above the reference value (25). Similarly, Starke et al. observed that in all 41 microbiologically or clinically documented infections, the maximum CRP level exceeded 30 mg/L, and in 25 cases, it was greater than 100 mg/L (26). Furthermore, Sato et al. demonstrated that high-sensitivity CRP measurement enables the detection of slight increases in serum CRP levels, which may reflect minute inflammation caused by occult infection, thereby identifying high-risk patients for infectious events (27). These prior observations are in concordance with our current findings.

Based on these features, we developed a predictive model integrating BSA and hs-CRP to assist clinical decision-making. The model offers three major advantages: first, it improves early diagnostic accuracy, particularly in children with lower BSA and hs-CRP; second, it guides rational antibiotic use—when the predicted probability exceeds 80%, empirical broad-spectrum antibiotic therapy may be deferred, and diagnosis confirmed through close clinical observation within 48 h, thereby reducing unnecessary antibiotic exposure and associated adverse effects; third, it provides real-world evidence to inform updates to pediatric drug labeling.

A standardized clinical management pathway is proposed: initial collection of core variables for risk assessment, followed by risk-stratified management (low risk: <20%; intermediate risk: 20%–80%; high risk: >80%). For patients stratified to intermediate- or high-risk groups, initial treatment with narrower-spectrum empiric antibiotics may be considered pending microbiological culture results. Importantly, any decision regarding antibiotic de-escalation should be implemented under rigorous clinical monitoring. Nevertheless, the generalizability and predictive performance of this model require further validation through multicenter prospective studies. Incorporation of more specific biomarkers (e.g., IL-6) may further enhance its discriminative ability.

Several limitations must be acknowledged. First, this study is a single-center retrospective study, with a relatively limited sample size. Therefore, the predictive model still needs to be further validated for its accuracy and clinical application effectiveness in multiple centers with large samples. Second, although the predictive model's efficacy was tested through internal validation methods in this study, its effectiveness should be further validated through external validation of the dataset. Finally, the mechanistic relationship between BSA, drug metabolism, and cytokine release remains incompletely elucidated and warrants further investigation. Future multicenter studies should validate the model, ideally with stepped-wedge designs investigating the predictive value of inflammatory markers, developing embedded electronic medical record alert systems, promoting the formulation of pediatric-specific management guidelines, and strengthening the integration of antimicrobial stewardship (AMS) and pharmacovigilance systems. Short-term efforts should focus on refining the CS-related fever diagnostic workflow; medium-term goals include establishing a multicenter network for monitoring adverse drug reactions in children; and long-term objectives should involve advancing a comprehensive drug evaluation system for pediatric therapeutics.

## Conclusion

This study successfully developed and validated a clinical prediction model for identifying cytarabine syndrome-related fever in pediatric patients with acute lymphoblastic leukemia. By integrating BSA and hs-CRP into a multivariate logistic regression framework, a user-friendly nomogram was constructed. Validation demonstrated that the model exhibits good discriminative performance and good calibration, with decision curve analysis confirming its ability to provide significant net benefit across a wide range of threshold probabilities. This tool enables the early and quantitative identification of cytarabine syndrome, thereby assisting clinicians in implementing evidence-based antibiotic stewardship for febrile episodes and avoiding unnecessary antimicrobial exposure. Future external validation and prospective refinement of the model will facilitate its broader clinical application, ultimately contributing to more standardized and personalized supportive care in pediatric leukemia treatment.

## Data Availability

The original contributions presented in the study are included in the article/Supplementary Material, further inquiries can be directed to the corresponding author.
